# Geographical variation of diabetic emergencies attended by prehospital Emergency Medical Services is associated with measures of ethnicity and socioeconomic status

**DOI:** 10.1038/s41598-018-23457-5

**Published:** 2018-03-23

**Authors:** Melanie Villani, Arul Earnest, Karen Smith, Barbora de Courten, Sophia Zoungas

**Affiliations:** 1 Monash Centre for Health Research and Implementation – MCHRI, School Public Health and Preventive Medicine, Monash University in partnership with Monash Health, 43-51 Kanooka Grove, Clayton, Victoria, 3168 Australia; 2 Research and Evaluation, Ambulance Victoria, 375 Manningham Road, Doncaster, Victoria, 3108 Australia; 3 Department of Epidemiology and Preventive Medicine, School of Public Health and Preventive Medicine, Monash University, Alfred Hospital, Commercial Road, Victoria, 3004 Australia; 4 Diabetes and Vascular Medicine Unit, Monash Health, 246 Clayton Road, Clayton, Victoria, 3168 Australia; 5 Department of Community Emergency Health and Paramedic Practice, School of Primary and Allied Health Care, Frankston, Victoria, 3199 Australia

## Abstract

Geographical variation of diabetic emergencies attended by prehospital emergency medical services (EMS) and the relationship between area-level social and demographic factors and risk of a diabetic emergency were examined. All cases of hypoglycaemia and hyperglycaemia attended by Ambulance Victoria between 1/01/2009 and 31/12/2015 were tabulated by Local Government Area (LGA). Conditional autoregressive models were used to create smoothed maps of age and gender standardised incidence ratio (SIR) of prehospital EMS attendance for a diabetic emergency. Spatial regression models were used to examine the relationship between risk of a diabetic emergency and area-level factors. The areas with the greatest risk of prehospital EMS attendance for a diabetic emergency were disperse. Area-level factors associated with risk of a prehospital EMS-attended diabetic emergency were socioeconomic status (SIR 0.70 95% CrI [0.51, 0.96]), proportion of overseas-born residents (SIR 2.02 95% CrI [1.37, 2.91]) and motor vehicle access (SIR 1.47 95% CrI [1.08, 1.99]). Recognition of areas of increased risk of prehospital EMS-attended diabetic emergencies may be used to assist prehospital EMS resource planning to meet increased need. In addition, identification of associated factors can be used to target preventative interventions tailored to individual regions to reduce demand.

## Introduction

Diabetes is associated with considerable healthcare resource use^1^, which may extend to prehospital Emergency Medical Services (EMS) when potentially life-threatening events such as severe hypoglycaemia or hyperglycaemia are experienced^2,3,4^. The geographical distribution of emergency medical events may occur in predictable patterns and may be related to specific characteristics of an area, such as socioeconomic status, remoteness, population density and ethnic composition. Spatial analysis and Geographical Information Systems (GIS) have previously been used to link area-level factors such as socioeconomic status and population density with medical-related ambulance requests^5^, as well as incidence and outcomes of acute myocardial infarction and cardiac arrest^6,7,8^. Similarly, geographical variation in environmental and socioeconomic factors is thought to contribute to variation in diabetes prevalence^9^ and incidence^10^, glycaemic control^11^ and prevalence of diabetes complications^12,13^.

The geographical distribution of prehospital EMS attendance to diabetic emergencies has not been previously reported. The benefit of geographical analysis of these events is two-fold. First, recognition of areas of increased demand may assist prehospital EMS resource planning to meet increased need and identify areas where access to services may be improved in order to reduce demand^14^. Second, description of the influence of area-level environmental and socioeconomic factors may assist in risk prediction, allowing identification of areas where targeted interventions would be most beneficial. The aims of this project were to examine the geographical distribution of diabetic emergencies throughout one of the most populous states of Australia, Victoria, and the relationship between area-level sociodemographic factors and risk of a diabetic emergency.

## Methods

### Study design

A state-wide, observational study of all prehospital EMS-attended diabetic emergencies between 1/01/2009 and 31/12/2015 was conducted. Individuals of all ages receiving emergency assistance from Ambulance Victoria, with a documented final primary assessment of hyperglycemia or hypoglycaemia were included. The Monash Health Human Research Ethics Committee approved this study [Project Approval Number 12197Q] and the study was performed in accordance with the guidelines and regulations of Monash Health and Monash University.

### Setting

At December 2015 the state of Victoria in Australia had an estimated residential population of 5.996 million^15^, with 300,400 residents registered with diabetes^16^. Ambulance Victoria is a two-tiered, prehospital EMS system and the sole provider of prehospital emergency medical care in the state. Geographically, Victoria is comprised of 79 Local Government Areas (LGAs), ranging in population from 3,085 to 267,640 residents and in size from 861.8 to 220,8248.3 ha^17^. Thirty-one LGAs make up Greater Melbourne, the broader metropolitan area in which the majority (75%) of the Victorian population, 4.529 million people, reside^18^. In addition to the 79 LGAs, a collection of 10 geographically distributed locations with migratory populations (islands and ski resorts) are administered under “Victoria unincorporated”. For the purpose of the current study, cases arising from unincorporated locations were excluded.

### Data Sources

Data were obtained from four sources; the Ambulance Victoria data warehouse, the Australian Bureau of Statistics (ABS), the National Diabetes Service Scheme (NDSS) and the Public Health Information Development Unit (PHIDU).

The AV data warehouse is an integrated data warehouse containing electronic patient care records and computer aided dispatch data for every case attended by Ambulance Victoria. Data regarding location of attendance, the final primary assessment and patient age, gender and diabetes type were extracted. The location of attendance was classified according to LGA. Diabetes type was based on self- or bystander reports and classified as type 1 diabetes, type 2 diabetes or unreported diabetes type/status. The final primary assessment, assigned by the attending paramedic, was defined as the main problem at the time the patient was discharged from EMS care and classified as hypoglycaemia or hyperglycaemia. No blood glucose level threshold parameters were imposed however, AV uses the BGL < 4 mmol/L to treat for hypoglycaemia^19^. Cases where the location of attendances was missing or located outside Victoria were excluded. Approximately 3 months of data [26/9/2014–20/12/2014] during the study period were unavailable due to lapse in electronic data collection. De-identified data was used with no ability to distinguish repeat callers, thus each attendance was considered as an individual case.

Population data regarding estimated residential population, population density (persons/km^2^), remoteness (Accessibility Remoteness Index for Australia, ARIA) and socioeconomic measures (socioeconomic indexes for areas, SEIFA) were obtained from the ABS^20^. Information on the percentage of residents with diabetes within each LGA was obtained from the NDSS^16^ and information regarding ethnicity and motor vehicle access was obtained from the PHIDU^21^.

### Covariates

The following area-level factors were examined: (1) socioeconomic disadvantage, (2) socioeconomic advantage and disadvantage, (3) economic resource, (4) education level and occupation, (5) remoteness, (6) population density, (7) prevalence of diabetes, (8) motor vehicle access and (9) proportion of overseas-born residents. For analysis, data for each covariate was divided into five categories, calculated by quintiles (except remoteness, which had 6 categories).

### Socioeconomic Indexes for Areas (SEIFA)

The SEIFA are based on information from the five-yearly census and include four indices that rank areas according to relative socioeconomic advantage and disadvantage. The *Index of Relative Socioeconomic Disadvantage* (ISRD) ranks areas on a continuum from most disadvantaged to least disadvantaged, the *Index of Relative Socioeconomic Advantage and Disadvantage* (ISRAD) ranks areas on a continuum from most disadvantaged to most advantaged, the *Index of Economic Resources* (IER) includes indicators of high and low income as variables that correlate to high or low wealth and the *Index of Education and Occupation* (IEO) includes the educational and occupation aspects of relative socioeconomic status and includes both the formal education and occupation-specific skills of the people of an area. The indices represent the collective socioeconomic characteristics of the people of an area. The categories for each SEIFA variable are as follows: IRSD; 1) <957.20 (most disadvantage), 2) 957.20 to <983.35, 3) 983.35 to <999.52, 4) 999.52 to <1037.75 and 5) ≥1037.75 (least disadvantage). IRSAD; 1) <942.54 (most disadvantage), 2) 942.54 to <969.86, 3) 969.86 to <993.08, 4) 993.08 to <1038.98 and 5) ≥1038.98 (most advantage). IEO; 1) <947.79 (lowest education), 2) 947.79 to <967.02, 3) 967.02 to <993.50, 4) 993.50 to <1050.63 and 5) ≥1050.63 (highest education). IER; 1) <963.79 (least wealth), 2) 963.79 to <975.75, 3) 975.75 to <1000.22, 4) 1000.22 to <1024.47 and 5) ≥1024 .47 (most wealth).

### Remoteness

The ARIA is a geographical approach to classifying the remoteness of an area derived from measures of distances between populated localities and five classes of service centre [ranging from 1,000–4,999 persons to < 250,000 persons]^22^. An ARIA category; Major City, Inner Regional, Outer Regional, Remote, Very Remote and Migratory/Offshore, is assigned to a Statistical Area (as distinct from an LGA). However, owing to Statistical Areas being smaller than LGAs, many LGAs correspond to two SAs and thus correspond to two ARIA categories. In addition, Victoria is geographically small compared to other Australian states and does not have any *Very Remote* or *Migratory/Offshore* areas, thus 6 categories; 1) “Major City”, 2) “Major City and Inner Regional”, 3) “Inner Regional”, 4) “Inner Regional and Outer Regional”, 5) “Outer Regional” and 6) “Outer Regional and Remote”, were used. The terminology “metropolitan” refers to Greater Melbourne and broadly corresponds to categories: 1 and 2, and “regional” refers to areas outside Greater Melbourne and broadly corresponds to categories 3 to 6.

### Population density

Population density (people per km^2^) was categorised; 1) <2.9 people/km^2^ (very low population density), 2) 2.9 to <8.4 people/km^2^, 3) 8.4 to <145.1 people/km^2^, 4) 145.1 to <1641.9 people/km^2^ and 5) ≥1641.9 people/km^2^ (very high population density).

### Prevalence of diabetes

The percentage of residents with diabetes was categorised; 1) <4.6% (low percentage of residents with diabetes), 2) 4.6 to <5.2%, 3) 5.2 to <5.7%, 4) 5.7 to <6.5% and 5) ≥6.5% (high percentage of residents with diabetes).

### Motor vehicle access

The percentage of dwellings with access to a motor vehicle was categorised; 1) >95.8% (high percentage of dwellings with access to a motor vehicle), 2) 95.8 to >94.4%, 3) 94.4 to >93.3%, 4) 93.3 to >91.9% and 5) ≤91.9 (low percentage of dwellings with access to a motor vehicle).

### Ethnicity

Ethnic composition of an area was represented by percentage of residents born overseas and categorised; 1) <12.3% (low percentage of residents born overseas), 2) 12.3 to <15.9%, 3) 15.9 to <20.5%, 4) 20.5 to <37.1% and 5) ≥37.1% (high percentage of residents born overseas).

### Statistical analysis

For each LGA, the observed caseload, observed case rate (per 10,000 residents) and expected caseload (age and gender standardised) were calculated. The observed caseload was calculated by tabulating the total number of diabetic emergency cases for each LGA. The observed case rate (per 10,000 residents) was calculated by dividing the total number of diabetic emergency cases for each LGA by the estimated residential population (ERP) of each LGA (for the middle study year, 2012) and multiplying by 10,000. The caseload of each LGA was indirectly standardised for age and gender using the state of Victoria as the standard population. Initially, cases (state-wide) were stratified by age and gender into 18 groups; males and females [0–9, 10–19, 20–29, 30–39, 40–49, 50–59, 60–69, 70–79, 80 + years]. The caseload of each age-gender group was divided by the Victorian ERP for the corresponding age-gender group, producing a standard population rate (SR) for each age-gender group. In the second step, the population of each LGA was stratified by age and gender, multiplied by the SR and summed to produce an *expected* caseload. This process was repeated separately for cases of hypoglycaemia and hyperglycaemia.

### Standardised Incidence Ratio (SIR)

The standardised incidence ratio (SIR), a measure of area-level disease risk, is defined as the *ratio of observed against expected cases in each LGA*. The crude SIR was calculated for each LGA and was repeated separately for cases of hypoglycaemia and hyperglycaemia. While the crude SIR provides a simple measure area-level disease risk, it may be obscured by sampling variability and imprecise for areas with small population. Geographical correlation in outcome is also expected in the data. Therefore, we have undertaken Bayesian spatial hierarchical modelling as way of distinguishing true risk variation from random noise and thus providing a more reliable estimate. LGAs sharing a common boundary were considered neighbours and adjacency was assigned using the Queen method of adjacency and all neighbours provided equal weights. This adjacency information is used by the model to determine the level of spatial correlation inherent in the data. The median number of neighbours of Victorian LGAs was five, and ranged from one to nine.

Bayesian hierarchical modelling with a conditional autoregressive (CAR) prior distribution was used to model the distribution of diabetic emergencies. The CAR distribution is represented as:

1 $$\begin{array}{c}{O}_{i} \sim {\rm{Poi}}({\mu }_{i}),\\ \mathrm{log}\,{\mu }_{i}=\,\mathrm{log}(Ei)+{u}_{i}+{v}_{i}\end{array}$$

where *Oi* and *Ei* are observed and expected caseload of the *i*^*th*^ LGA, respectively *u*_*i*_ is a spatially structured random effect that is assigned a CAR prior distribution and *v*_*i*_ is a spatially unstructured random effect^5^. Where various factors related to risk of a diabetic emergency were modelled, univariable and multivariable models included the addition of coefficients in the above model.

Bayesian hierarchical modelling was carried out with two chains and with uninformative priors. The initial 100,000 estimates were discarded (burn in) and a further 500,000 iterations were performed. Every other iterated value was selected, to remove autocorrelated samples. Convergence was assessed with the Gelman-Rubin convergence statistic as well as visual inspection of the Brooks-Gelman-Rubin (BGR) graph. The deviance information criterion (DIC) was used to assess the complexity and fit of the models (where smaller DIC values are preferred).

We examined the influence of nine area-level factors (the four SEIFA indices, remoteness, population density, prevalence of diabetes, access to a motor vehicle and ethnic composition) on the incidence ratio of prehospital EMS-attended diabetic emergencies. The standardised incidence ratio accounts for age and gender, thus these variables were not additionally included in the modelling. Initially, univariable analysis was performed for each variable separately. The multivariable modelling was performed in a stepwise manner, whereby factors were included sequentially (in order of lowest DIC obtained in the univariable analysis) and retained in the model when a significant result was observed (i.e. the 95% CrI did not include zero). Given the overlapping theme of the four SEIFA indices, one variable (IRSD) was selected to represent socioeconomic status in the multivariable modelling and IEO, IRSAD and IER were omitted. This process was repeated for cases of hypoglycaemia and hyperglycaemia separately.

Data analysis was performed in Stata 14.0 and WinBUGS 14. Adjacency was determined using GeoDa 1.8.16.4 and visual maps representing SIR were produced in ArcMap version 10.4.1 (Esri)^23^. The datasets analysed during the current study are available from the corresponding author upon reasonable request.

## Results

Ambulance Victoria attended 41,454 diabetic emergencies (cases of hypoglycaemia and hyperglycaemia) during the 7-year study period, of which 39,332 contained sufficient GPS location data for inclusion. Of these 69.9% (n = 27,483) were cases of hypoglycaemia and 30.1% (n = 11,849) were cases of hyperglycaemia. Males accounted for 55.4% of cases (n = 21,789) and the median [IQR] age was 59 [40, 76] years with a skewed distribution towards older-age patients (Supplimentary appendix Figure S1).

### Observed and expected caseload and Standardised Incidence Ratio

The observed caseload, observed case rate per 10,000 residents, expected caseload, and standardised incidence ratio (SIR) for a) combined diabetic emergencies, b) hypoglycaemia and c) hyperglycaemia for each LGA are reported in Supplementary Appendix 1 (Tables 3–5).

### Combined diabetic emergencies

**Figure 1:**
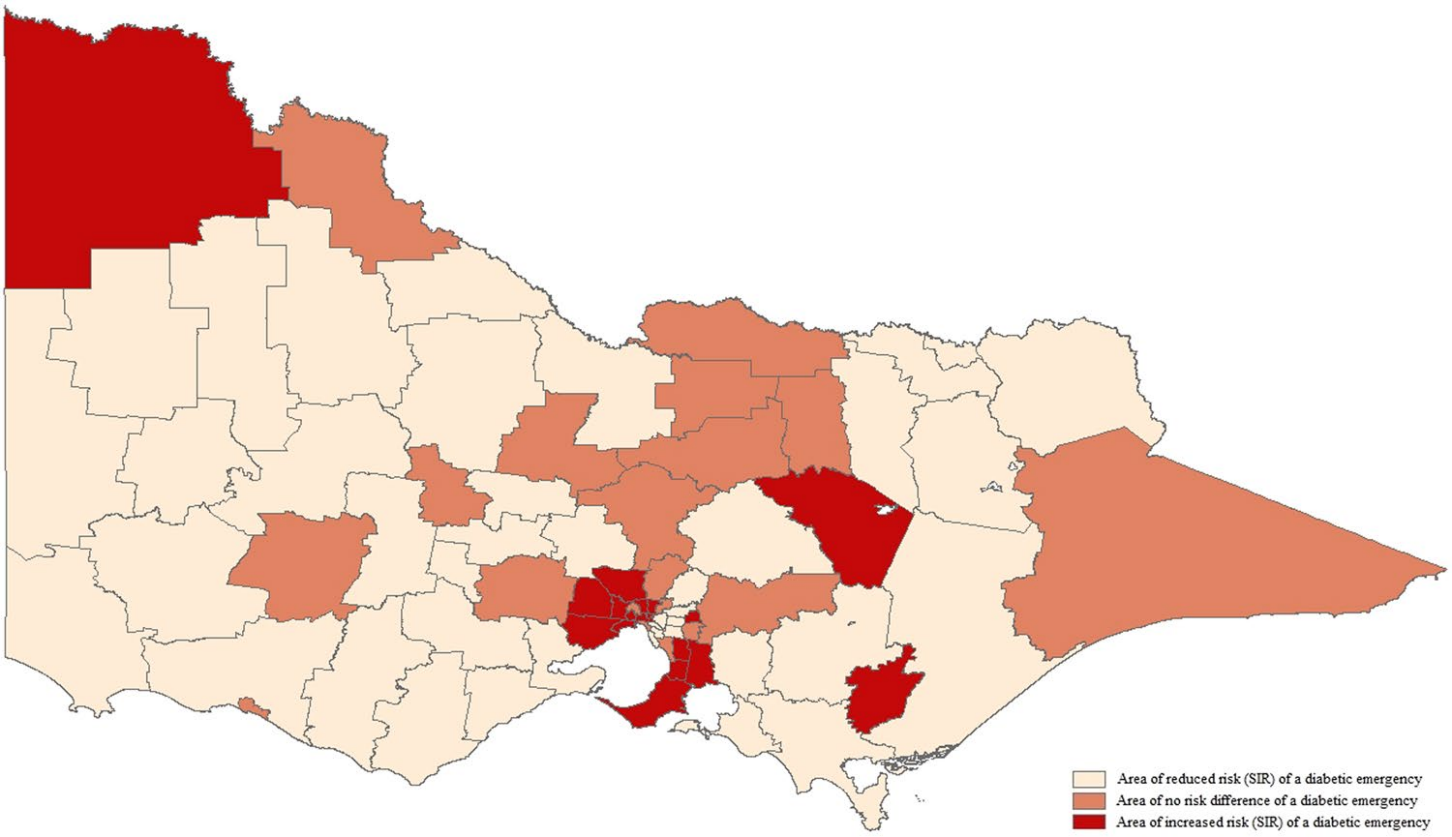
Standardised Incidence Ratio of prehospital EMS attendance for a diabetic emergency. Map generated in Esri ArcMap version 10.4.1^23^.

The observed case rates of prehospital EMS-attended diabetic emergencies (combined hypoglycaemia and hyperglycaemia) among the LGAs ranged from 9.54 to 132.89 per 10,000 residents. The areas with the highest observed case rates were disperse and included regional North-East and South-East, as well as outer-metropolitan South East and inner central metropolitan regions. Eighteen LGAs were identified as having increased risk (SIR) of prehospital EMS-attended diabetic emergency (Fig. 1). The areas with the greatest risk of a prehospital diabetic emergency were inner central (SIR 1.77, 95% CrI [1.63, 1.92]), regional North-East (SIR 1.70, 95% CrI [1.32, 1.96]), outer-metropolitan South-East (SIR 1.55 95% CrI [1.44, 1.67], SIR 1.40, 95% CrI [1.29, 1.50]) and regional South-East (SIR 1.39 95% CrI [1.27, 1.52]).

### Hypoglycaemia

**Figure 2:**
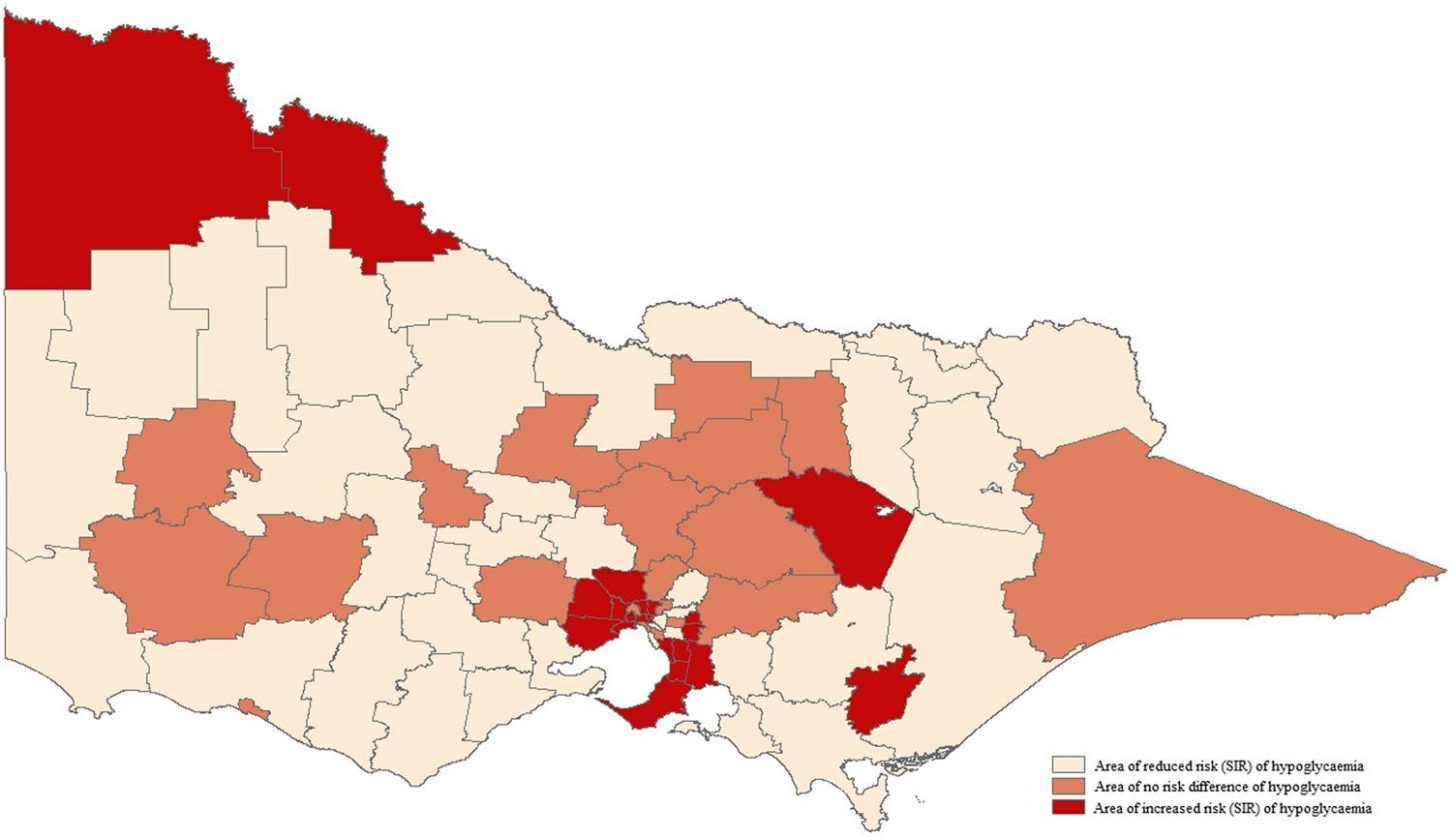
Standardised incidence ratio of prehospital EMS attendance for hypoglycaemia. Map generated in Esri ArcMap version 10.4.1^23^.

The observed case rate of prehospital EMS-attended hypoglycaemia among LGAs ranged from 4.77 to 103.08 per 10,000 residents. Areas with the highest case rates included regional North-East, inner central, outer-metropolitan South-East and remote Western regions. Twenty-one LGAs were identified as having increased risk of prehospital-attended hypoglycaemia (Fig. 2). Areas with the greatest risk of hypoglycaemia were geographically disperse and included inner central (SIR 1.91 95% CrI [1.77, 2.03]), regional North-East (SIR 1.75 95% CrI [1.40, 2.15]), outer metropolitan South-East (SIR 1.48 95% CrI [1.38, 1.57]), inner metropolitan East (SIR 1.47, 95% CrI [1.34, 1.60]) and inner metropolitan Western areas (SIR 1.45, 95% CrI [1.32, 1.58]).

### Hyperglycaemia

**Figure 3:**
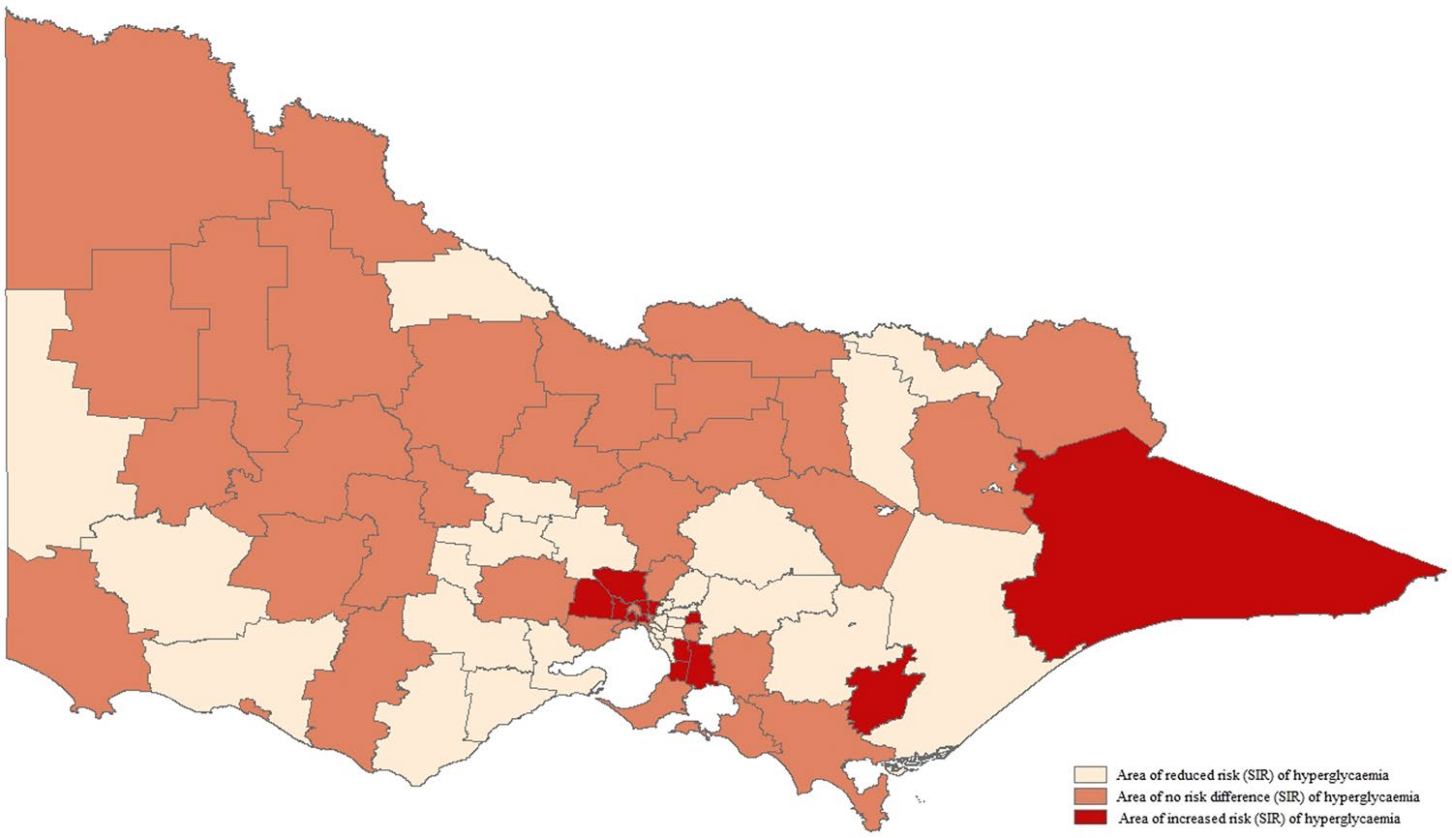
Standardised incidence ratio of prehospital EMS attendance for hyperglycaemia. Map generated in Esri ArcMap version 10.4.1^23^.

The observed case rate of prehospital EMS-attended hyperglycaemia among the LGAs ranged from 4.77 to 38.38 per 10,000 residents. Areas with the highest case rate of hyperglycaemia included regional South-East, outer metropolitan South-East and regional South-West. Thirteen LGAs were identified as having increased risk of hyperglycaemia (Fig. 3) and included inner regional and outer-metropolitan areas, spreading from the South-East (SIR 1.76 95% CrI [1.54, 1.99], SIR 1.72 95% CrI [1.54, 1.91], SIR 1.55 95% CrI [1.39, 1.72]) to the North-West (SIR 1.57 95% CrI [1.38,1.78], SIR 1.51 95% CrI [1.35,1.67]).

### Factors related to risk of a prehospital EMS-attended diabetic emergency

Factors associated with risk of a prehospital EMS-attended diabetic emergency are reported in Table 1 (unadjusted) and Table 2 (multivariable). Separate analyses of area-level factors associated with hypoglycaemia and hyperglycaemia are reported in the Supplementary appendix (Tables 6–9). Model convergence was deemed successful as the MC error was less than 5% of the standard deviation in all cases.

**Table 1:** Factors associated with risk of prehospital EMS attendance for a diabetic emergency: Unadjusted models.

Area-level factor	SIR	95% CrI	DIC
**Ethnicity (percentage of oversea-born residents)**			747.9
1 (low proportion overseas-born residents)	Reference		
2	1.31	[0.97, 1.75]	
3	1.5	[1.11, 2.05]*	
4	1.76	[1.25, 2.48]*	
5 (high proportion overseas-born residents)	2.02	[1.42, 2.87]*	
**Access to motor vehicle**			
1 (most access to motor vehicle)	Reference		753.0
2	1.13	[0.87, 1.47]	
3	1.14	[0.87, 1.48]	
4	1.55	[1.18, 2.01]*	
5 (least access to motor vehicle)	1.55	[1.17, 2.06]*	
**IEO (education and occupation)**			
1 (least education)	Reference		754.0
2	0.85	[0.64, 1.11]	
3	0.83	[0.64, 1.08]	
4	0.61	[0.47, 0.81]*	
5 (most education)	0.6	[0.43, 0.85]*	
**IRSD (socioeconomic disadvantage)**			
1 (most disadvantage)	Reference		754.8
2	0.76	0.58, 1.00]	
3	0.79	[0.60, 1.05]	
4	0.72	[0.54, 0.98]*	
5 (least disadvantage)	0.61	[0.43, 0.86]*	
**IRSAD (socioeconomic advantage & disadvantage)**			755.0
1 (most disadvantage)	Reference		
2	0.8	[0.60, 1.06]	
3	0.76	[0.57, 1.00]	
4	0.64	[0.47, 0.88]*	
5 (most advantage)	0.6	[0.42, 0.84]*	
**IER (economic resource)**			755.1
1 (least wealth)	Reference		
2	0.84	[0.64, 1.10]	
3	0.73	[0.55, 0.96]*	
4	0.76	[0.58, 0.99]*	
5 (most wealth)	0.57	[0.43, 0.75]*	
**ARIA (remoteness)**			758.2
1 (major city)	Reference		
2	0.91	[0.67, 1.22]	
3	0.69	[0.49, 0.95]*	
4	0.63	[0.44, 0.90]*	
5	0.86	[0.55, 1.39]	
6 (remote)	0.66	[0.39, 1.12]	
**Population density (residents per km** ^2^ **)**			
1 (low density)	Reference		743.1
2	0.86	[0.65, 1.16]	
3	1.03	[0.75, 1.41]	
4	1.4	[0.98, 2.00]	
5 (high density)	1.33	[0.90, 1.98]	
**Prevalence of diabetes (percentage residents with diabetes)**			754.8
Q1 (low prevalence)	Reference		
Q2	0.84	[0.60, 1.16]	
Q3	0.99	[0.74, 1.32]	
Q4	1.07	[0.78, 1.47]	
5 (high prevalence)	1.14	[0.83, 1.57]	

^*^Indicates statistical significance (i.e. 95% CrI does not cross 0).

**Table 2:** Factors associated with risk of prehospital EMS attendance for a diabetic emergency: Multivariable model.

Area-level factor	SIR	95% CrI	DIC
			742.4
**Ethnicity (percentage of oversea-born residents)**			
1 (low proportion overseas-born residents)	Reference		
2	1.29	0.97, 1.72]	
3	1.6	[1.18, 2.18]*	
4	1.86	[1.27, 2.65]*	
5 (high proportion overseas-born residents)	2.02	[1.37, 2.91]*	
**Access to motor vehicle**			
1 (most access to motor vehicle)	Reference		
2	1.11	[0.84, 1.46]	
3	1.15	[0.83, 1.58]	
4	1.47	[1.08, 1.99]*	
5 (least access to motor vehicle)	1.3	[0.97, 1.75]	
**IRSD (socioeconomic disadvantage)**			
1 (most disadvantage)	Reference		
2	0.89	[0.68, 1.16]	
3	0.91	[0.68, 1.22]	
4	0.89	[0.63, 1.24]	
5 (least disadvantage)	0.7	[0.51, 0.96]*	

*Indicates statistical significance (i.e. 95% CrI does not cross 0).

### Socioeconomic indices: IRSD, IRSAD, IEO, IER

Increased socioeconomic status was associated with reduced risk of prehospital EMS attendance for a diabetic emergency. Across the four indices, LGAs in the most advantaged category had approximately 40% lower risk of prehospital EMS attendance for a diabetic emergency when compared to the most disadvantaged category [IRSD (SIR 0.61, 95% CrI [0.43, 0.86]), IRSAD (SIR 0.60, 95% CrI [0.42, 0.84]), IEO (SIR 0.60, 95% CrI [0.43, 0.85]), IER (SIR 0.59, 95% CrI [0.41, 0.85]). In the multivariable model, IRSD (index of socioeconomic disadvantage) remained significantly associated, whereby areas in the most advantaged category had 30% reduced risk of prehospital EMS attendance for a diabetic emergency (SIR 0.70, 95% CrI [0.51, 0.96]) (Table 2).

### Remoteness

In the univariable analysis, inner-regional and inner-to-outer-regional locations had reduced risk of prehospital EMS attendance for diabetic emergency when compared to major city areas (SIR: 0.69, 95% CrI [0.49, 0.95] and SIR: 0.63, 95% CrI [0.44, 0.90] respectively), however, this association did not remain significant in the multivariable analysis.

### Ethnicity

Areas with the highest and second highest proportion of overseas born residents had a 102% (SIR 2.02, 95% CrI [1.42, 2.87]) and 76% (SIR 1.76, 95% CrI [1.25, 2.48]) increased risk of prehospital EMS attendance for a diabetic emergency, respectively. In the multivariable analysis, this association remained significant and occurred in a dose-dependent fashion whereby as the proportion of overseas-born residents increased, the risk of prehospital EMS-attended diabetic emergency increased by approximately 20% per quintile (SIR 1.60 95% CrI [1.18, 2.18], SIR 1.86 95% CrI [1.27, 2.65], SIR 2.02 95% CrI [1.37, 2.91] in the top three quintiles, respectively).

### Motor vehicle access

Compared to areas with the greatest access to a motor vehicle, areas in the lowest two categories had a 55% increased risk of prehospital EMS attendance for a diabetic emergency (SIR 1.55, 95% CrI [1.18, 2.01]) (SIR 1.55, 95% CrI [1.17, 2.06]). In the multivariable model, areas in the second lowest category of motor vehicle access (93.3–91.9%) had a 47% increased risk of EMS attendance for a diabetic emergency compared to areas with greater access to a motor vehicle (SIR 1.47 95% CrI [1.08, 1.99]).

### Population density and prevalence of diabetes

There was no significant risk difference associated with population density nor proportion of population with diabetes and prehospital attendance for a diabetic emergency.

Individual analyses of EMS attendances for hypoglycaemia and hyperglycaemia revealed differences in area-level factors related to risk in the multivariable models. While risk of hypoglycaemia and hyperglycaemia were both associated with ethnicity, (a 104% increased risk of hypoglycaemia and a 90% increased risk of hyperglycaemia) in areas with the greatest proportion of overseas born residents, (SIR 2.04 95%Crl [1.39, 2.91], SIR 1.90 95% CrI [1.27, 2.80]), risk of hypoglycaemia was also associated with access to a motor vehicle (a 38% increased risk in areas with least access to a motor vehicle, SIR 1.38 95% CrI [1.04, 1.83]), and hyperglycaemia was also associated with socioeconomic status (a 45% reduced risk in areas of least disadvantage, SIR 0.55 95% CrI [0.43, 0.91]).

## Discussion

We have shown considerable regional variation in prehospital EMS-attended diabetic emergencies across the state of Victoria, Australia and identified areas of increased utilisation of prehospital EMS for diabetic emergencies. When examined separately, areas of increased risk of hyperglycaemia and hypoglycaemia demonstrated some overlap (outer metropolitan West and outer metropolitan South East), however there were more areas of increased risk of hypoglycaemia in the remote West while the remote East tended to contain increased risk of hyperglycaemia. We have demonstrated an application of geospatial analysis in describing the relationship between population factors and diabetic emergencies. The areas and factors related to increased risk of a diabetic emergency described in this paper may assist prehospital EMS systems and health services with future planning of limited resources.

Area-level characteristics independently associated with increased risk of a prehospital EMS-attended diabetic emergency were proportion of overseas born residents, socioeconomic status and access to a motor vehicle. Surprisingly, population density and prevalence of diabetes were not associated with the risk of a prehospital EMS-attended diabetic emergency and remoteness appeared to be accounted for by the other factors. When examined by type of emergency, both hypoglycaemia and hyperglycaemia were associated with proportion of overseas born residents, however, hypoglycaemia was also associated with access to a motor vehicle and hyperglycaemia was also associated with socioeconomic status.

This is the first study to demonstrate an association between socioeconomic status and risk of a prehospital diabetic emergency at an ecological level, finding a 30% reduced risk in the most advantaged areas compared to the most disadvantaged. This is in line with previous literature on socioeconomic health inequalities^24,25^ and the social gradient of health^26^ and, in particular, diabetes prevalence^27^ and complications^28,29^. Furthermore, we found a strong dose-dependent association between area-level ethnic composition (proportion of overseas born residents) and risk of prehospital diabetic emergency, independent of socioeconomic disadvantage. In support, a multifactorial and complex relationship between race, socioeconomic status^30^ and health, including factors such as migratory history, genetics^31^ and health literacy^32^, has been proposed. While the current study didn’t have access to detailed-enough information to isolate particular ethnicities at risk, findings may be used to target preventive interventions across areas with high linguistic and cultural diversity.

Areas with lower motor vehicle access were more likely to have a prehospital EMS-attended diabetic emergency. Previous literature regarding associations between motor vehicle access and utilisation of the prehospital EMS is scarce^33^. The current study included all cases of hypoglycaemia and hyperglycaemia for which an ambulance attended but could not make a distinction regarding severity. A possible explanation could be that patients without a motor vehicle may have a propensity to seek prehospital EMS care rather than general practitioner care when ‘mildly unwell’.

The association between remoteness and a prehospital EMS-attended diabetic emergency observed in the univariable analysis was not seen in the multivariable model, suggesting it was accounted for by ethnic, socioeconomic and vehicle access differences. The relationship between remoteness and prehospital EMS utilisation is unclear and a range of factors including differences in rates of pre-diabetes and uncontrolled diabetes^34^, access to primary and specialist care, prehospital EMS-seeking behaviours^35^, as well as ethnic and indigenous composition between urban and non-urban areas may be involved. However, as Victoria is a smaller state that contains very few remote areas compared to other Australian states, an independent effect of remoteness cannot be completely excluded. Further research, examining the relationship between remoteness, diabetes, diabetes complications and prehospital EMS-seeking behaviours is required.

Interestingly, the prevalence of diabetes did not increase the risk of prehospital diabetic emergencies at an ecological level. This finding is difficult to explain and could be related to health seeking behaviours in urban and non-urban areas, or proximity to local services. Nonetheless, the findings may be useful for those targeting interventions aimed at reducing diabetic emergencies, whereby use of socioeconomic and ethnicity factors could be more useful than diabetes prevalence alone.

A strength of this study is that it captures all prehospital EMS-attended diabetic emergencies in Victoria, Australia. Our findings form the basis for further research, such as geographically targeted case-control studies between the highest and lowest risk areas to further evaluate risk factors, and evaluate interventions. It is unknown how many people with a diabetic emergency attended hospital directly, and thus the proportional burden on the prehospital EMS. However international work shows that of all ED presentations of hypoglycaemia, the majority (84%) were transported by EMS^36^ and that 86% of all cases of hypoglycaemia involved contact with prehospital EMS^29^.

The effect of proximity to local services of risk of prehospital EMS use for a diabetic emergency was outside the scope of this study and thus is a limitation of the current study and an interesting direction for future research. The missing three months of data and the use of de-identified data resulting in repeat attendances reported as individual cases, are also limitations. In addition, the measure of ethnicity used (percentage of overseas-born residents) does not account for Aboriginal status and does not examine the effects of specific ethnicities. As with any ecological study there is a risk that the resident population may not accurately reflect the population at risk, as people travel between regions for work and leisure. However, our previous work has indicated that the majority (approximately 80%) of prehospital EMS-attended hypoglycaemia and hyperglycaemia events occur at a private residence^3,4^. Furthermore, associations observed at the ecological level may not occur at the individual level.

We have demonstrated regional variation in prehospital EMS attendance for diabetic emergencies across a populous Australian state and identified associated area-level characteristics. These findings may assist prehospital EMS systems and health services in planning and appropriate resource provision. More research regarding further evaluation of targeted interventions to ease prehospital EMS demand is required.

## Electronic supplementary material

Supplimentary Appendix
