# Supplementary material for: Geographical variation of diabetic emergencies attended by prehospital Emergency Medical Services is associated with measures of ethnicity and socioeconomic status
Source: Sci Rep. 2018 Mar 23;8:5122. doi: 10.1038/s41598-018-23457-5 (PMC5865134; doi:10.1038/s41598-018-23457-5)
Supplement: Supplementary file 1 — Supplimentary Appendix [file 41598_2018_23457_MOESM1_ESM.pdf]

Supplementary Appendix

Geographical variation of diabetic emergencies attended by prehospital Emergency Medical Services is associated with measures of ethnicity and socioeconomic status

Melanie Villani[1,2,3], Arul Earnest[1,3], Karen Smith[2,3,5], Barbora de Courten[1,4] and Sophia Zoungas[1,3,4]

Affiliations

1. Monash Centre for Health Research and Implementation – MCHRI, School Public Health and Preventive Medicine, Monash University in partnership with Monash Health, 43 – 51 Kanooka Grove, Clayton, Victoria 3168, Australia.
2. Research and Evaluation, Ambulance Victoria, 375 Manningham Road, Doncaster, Victoria 3108, Australia.
3. Department of Epidemiology and Preventive Medicine, School of Public Health and Preventive Medicine, Monash University, Alfred Hospital, Commercial Road, Victoria 3004, Australia.
4. Diabetes and Vascular Medicine Unit, Monash Health, 246 Clayton Road, Clayton, Victoria 3168, Australia.
5. Department of Community Emergency Health and Paramedic Practice, School of Primary and Allied Health Care, Frankston, Victoria 3199, Australia.

Corresponding author

Professor Sophia Zoungas

Division of Metabolism, Genomics and Ageing,

Department of Epidemiology and Preventive Medicine,

School of Public Health and Preventive Medicine, Monash University

5th Floor, 99 Commercial Road

Melbourne, VIC 3004, Australia

Phone: +61 3 9903 0711

E-mail: sophia.zoungas@monash.edu

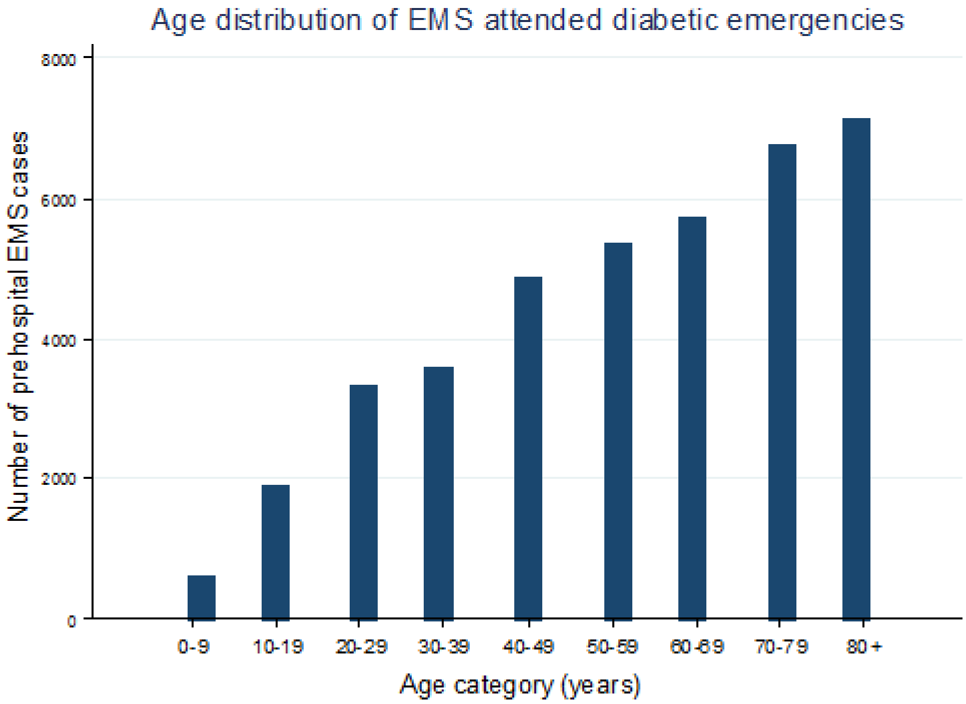

Figure S1: Age distribution of prehospital EMS attended diabetic emergencies

Table 3: Combined Diabetic Emergencies: Observed case rate, standardised case rate and Standardised Incidence Ratio (SIR)

LGA	*Observed caseload*	*Observed case rate (per 10,000)*	*Expected caseload*	*SIR (crude)*	*SIR [CrI] (smoothed)*
Alpine	68	56.16	102.70	0.66	0.67 [0.53, 0.83]
Ararat	104	91.75	93.13	1.12	1.08 [0.87, 1.30]
Ballarat	590	60.87	676.05	0.87	0.87 [0.79, 0.96]
Banyule	873	70.63	892.49	0.98	0.98 [0.90, 1.06]
Bass Coast	208	67.94	260.52	0.80	0.80 [0.69, 0.92]
Baw Baw	238	53.73	325.22	0.73	0.74 [0.64, 0.84]
Bayside	438	45.16	745.81	0.59	0.59 [0.53, 0.66]
Benalla	103	75.01	116.47	0.88	0.88 [0.72, 1.07]
Boroondara	794	47.13	1200.59	0.66	0.66 [0.61, 0.72]
Brimbank	1450	75.00	1235.54	1.17	1.17 [1.09, 1.26]
Buloke	27	42.49	57.34	0.47	0.52 [0.36, 0.70]
Campaspe	253	68.44	290.71	0.87	0.87 [0.76, 0.99]
Cardinia	410	51.13	485.17	0.85	0.85 [0.76, 0.94]
Casey	1935	72.30	1561.92	1.24	1.24 [1.15, 1.33]
Central Goldfields	102	81.07	112.22	0.91	0.89 [0.73, 1.08]
Colac-Otway	94	45.26	163.07	0.58	0.58 [0.47, 0.70]
Corangamite	84	51.26	129.11	0.65	0.65 [0.52, 0.79]
Darebin	1193	82.48	1010.87	1.18	1.18 [1.09, 1.28]
East Gippsland	379	87.81	370.54	1.02	1.02 [0.91, 1.14]
Frankston	1389	105.42	893.39	1.55	1.55 [1.44, 1.67]
Gannawarra	58	55.90	92.25	0.63	0.64 [0.49, 0.81]
Glen Eira	875	62.84	995.04	0.88	0.88 [0.81, 0.96]
Glenelg	114	57.70	152.89	0.75	0.73 [0.60, 0.88]
Golden Plains	41	21.06	123.59	0.33	0.37 [0.28, 0.48]
Gr. Bendigo	731	70.56	741.98	0.99	0.98 [0.90, 1.08]
Gr Dandenong	1371	95.00	981.86	1.40	1.40 [1.29, 1.50]
Gr. Geelong	1352	61.98	1616.47	0.84	0.84 [0.77, 0.90]
Gr. Shepparton	441	70.70	435.96	1.01	1.01 [0.90, 1.12]
Hepburn	46	31.23	118.44	0.39	0.42 [0.32, 0.54]
Hindmarsh	33	57.08	52.31	0.63	0.64 [0.45, 0.86]
Hobsons Bay	689	78.05	609.44	1.13	1.13 [1.03, 1.24]
Horsham	127	64.69	150.37	0.84	0.83 [0.69, 0.99]
Hume	1307	73.41	1035.86	1.26	1.26 [1.17, 1.36]
Indigo	43	27.97	117.60	0.37	0.40 [0.30, 0.52]
Kingston	1173	78.19	1097.01	1.07	1.07 [0.99, 1.16]
Knox	1137	73.55	1045.59	1.09	1.09 [1.00, 1.18]
Latrobe	739	99.86	530.29	1.39	1.39 [1.27, 1.52]
Loddon	47	62.62	67.70	0.69	0.70 [0.53, 0.91]
Macedon Ranges	210	48.26	297.72	0.71	0.71 [0.61, 0.82]
Manningham	597	51.01	919.08	0.65	0.65 [0.59, 0.72]
Mansfield	107	132.89	63.07	1.70	1.62 [1.32, 1.96]
Maribyrnong	665	86.51	476.98	1.39	1.39 [1.27, 1.53]
Maroondah	902	83.39	756.58	1.19	1.19 [1.09, 1.29]
Melbourne	1033	97.99	581.35	1.78	1.77 [1.63, 1.92]
Melton	844	71.60	626.55	1.35	1.34 [1.23, 1.46]
Mildura	477	91.38	379.96	1.26	1.25 [1.12, 1.38]
Mitchell	243	67.23	232.84	1.04	1.04 [0.90, 1.19]
Moira	209	72.96	238.37	0.88	0.87 [0.75, 1.01]
Monash	1098	61.02	1337.36	0.82	0.82 [0.76, 0.89]
Moonee Valley	839	73.91	817.82	1.03	1.03 [0.94, 1.12]
Moorabool	181	61.68	195.60	0.93	0.92 [0.79, 1.07]
Moreland	1372	87.58	1097.89	1.25	1.25 [1.16, 1.35]
Mornington	1410	93.48	1237.87	1.14	1.14 [1.06, 1.23]
Mount Alexander	67	37.44	150.70	0.44	0.47 [0.37, 0.58]

Moyne	63	20.10	587.79	0.11	0.13	[0.10, 0.16]
Murrindindi	89	66.30	106.22	0.84	0.84	[0.68, 1.03]
Nillumbik	271	43.19	391.58	0.69	0.70	[0.61, 0.79]
Nth Grampians	76	63.62	99.35	0.76	0.76	[0.60, 0.94]
Port Phillip	628	62.55	637.58	0.98	0.99	[0.89, 1.08]
Pyrenees	41	60.78	58.14	0.71	0.70	[0.52, 0.92]
Queenscliffe	25	81.04	31.74	0.79	0.79	[0.53, 1.11]
Sth Gippsland	145	52.24	223.10	0.65	0.66	[0.55, 0.77]
Sth Grampians	100	61.08	132.33	0.76	0.74	[0.60, 0.90]
Stonnington	535	52.87	709.80	0.75	0.76	[0.68, 0.83]
Strathbogie	83	85.87	88.29	0.94	0.94	[0.75, 1.15]
Surf Coast	98	35.67	191.29	0.51	0.52	[0.42, 0.63]
Swan Hill	174	83.05	152.97	1.14	1.12	[0.95, 1.30]
Towong	28	47.38	50.10	0.56	0.59	[0.41, 0.81]
Wangaratta	137	50.33	215.48	0.64	0.64	[0.54, 0.76]
Warrnambool	224	67.87	238.69	0.94	0.93	[0.80, 1.06]
Wellington	200	47.29	317.97	0.63	0.64	[0.55, 0.73]
West Wimmera	4	9.54	36.17	0.11	0.28	[0.16, 0.44]
Whitehorse	1068	67.04	1191.41	0.90	0.90	[0.83, 0.97]
Whittlesea	1061	62.43	1035.60	1.02	1.03	[0.94, 1.11]
Wodonga	203	55.50	235.57	0.86	0.86	[0.74, 0.99]
Wyndham	1074	60.05	950.85	1.13	1.13	[1.04, 1.22]
Yarra	689	85.08	504.12	1.37	1.36	[1.24, 1.49]
Yarra Ranges	972	65.13	994.16	0.98	0.98	[0.90, 1.06]
Yarriambiack	34	47.87	65.45	0.52	0.55	[0.39, 0.73]

Table 4: Hypoglycaemia: Observed case rate, standardised case rate and standardised incidence ratio

LGA	*Observed caseload*	*Observed case rate (per 10,000)*	*Expected caseload*	*SIR (crude)*	*SIR [CrI] (smoothed)*
Alpine	45	37.16	72.03	0.62	0.64 [0.48, 0.82]
Ararat	64	56.46	65.30	0.98	0.93 [0.72, 1.16]
Ballarat	426	43.95	471.12	0.90	0.90 [0.82, 0.99]
Banyule	640	51.78	622.87	1.03	1.03 [0.95, 1.11]
Bass Coast	145	47.36	182.32	0.80	0.80 [0.67, 0.93]
Baw Baw	188	42.44	227.77	0.83	0.83 [0.72, 0.95]
Bayside	296	30.52	519.15	0.57	0.58 [0.51, 0.64]
Benalla	69	50.25	81.23	0.85	0.85 [0.67, 1.05]
Boroondara	612	36.33	834.96	0.73	0.74 [0.68, 0.79]
Brimbank	967	50.02	866.95	1.12	1.12 [1.05, 1.19]
Buloke	8	12.59	39.95	0.20	0.34 [0.21, 0.51]
Campaspe	174	47.07	203.16	0.86	0.85 [0.73, 0.98]
Cardinia	279	34.79	340.41	0.82	0.82 [0.73, 0.92]
Casey	1237	46.22	1095.70	1.13	1.13 [1.07, 1.19]
Central Goldfields	66	52.46	78.42	0.84	0.82 [0.64, 1.03]
Colac-Otway	63	30.34	113.90	0.55	0.56 [0.43, 0.70]
Corangamite	47	28.68	90.28	0.52	0.53 [0.40, 0.68]
Darebin	762	52.68	705.42	1.08	1.08 [1.01, 1.16]
East Gippsland	238	55.14	259.65	0.92	0.91 [0.80, 1.03]
Frankston	925	70.21	625.15	1.48	1.48 [1.38, 1.57]
Gannawarra	46	44.33	64.34	0.71	0.72 [0.54, 0.92]
Glen Eira	656	47.11	692.32	0.95	0.95 [0.88, 1.02]
Glenelg	61	30.87	107.22	0.57	0.56 [0.44, 0.71]
Golden Plains	26	13.35	87.38	0.30	0.35 [0.25, 0.48]
Gr. Bendigo	491	47.39	517.84	0.95	0.95 [0.86, 1.03]
Gr Dandenong	912	63.20	686.46	1.33	1.33 [1.24, 1.41]
Gr. Geelong	979	44.88	1127.38	0.87	0.87 [0.81, 0.92]
Gr. Shepparton	316	50.66	304.71	1.04	1.03 [0.92, 1.15]
Hepburn	31	21.05	83.22	0.37	0.42 [0.30, 0.56]
Hindmarsh	22	38.06	36.36	0.61	0.61 [0.41, 0.86]
Hobsons Bay	500	56.64	426.91	1.17	1.17 [1.07, 1.28]
Horsham	89	45.34	104.74	0.85	0.83 [0.67, 1.01]
Hume	840	47.18	727.39	1.15	1.15 [1.08, 1.23]
Indigo	30	19.51	82.68	0.36	0.41 [0.29, 0.55]
Kingston	883	58.86	766.30	1.15	1.15 [1.08, 1.23]
Knox	794	51.36	732.58	1.08	1.08 [1.01, 1.16]
Latrobe	455	61.49	370.84	1.23	1.22 [1.11, 1.33]
Loddon	27	35.97	47.53	0.57	0.60 [0.42, 0.81]
Macedon Ranges	166	38.15	209.50	0.79	0.79 [0.68, 0.92]
Manningham	425	36.31	642.41	0.66	0.67 [0.60, 0.73]
Mansfield	83	103.08	44.29	1.87	1.75 [1.40, 2.15]
Maribyrnong	485	63.10	334.40	1.45	1.45 [1.32, 1.58]
Maroondah	636	58.80	527.96	1.20	1.20 [1.11, 1.30]
Melbourne	773	73.33	405.20	1.91	1.90 [1.77, 2.03]
Melton	550	46.66	441.20	1.25	1.24 [1.14, 1.35]
Mildura	340	65.14	265.11	1.28	1.26 [1.13, 1.40]
Mitchell	163	45.10	163.53	1.00	0.99 [0.85, 1.15]
Moira	129	45.03	166.48	0.77	0.77 [0.65, 0.91]
Monash	804	44.68	931.98	0.86	0.86 [0.81, 0.92]
Moonee Valley	596	52.50	570.69	1.04	1.05 [0.96, 1.13]
Moorabool	124	42.25	137.47	0.90	0.89 [0.75, 1.06]
Moreland	890	56.81	764.45	1.16	1.16 [1.09, 1.24]
Mornington	1028	68.16	863.20	1.19	1.19 [1.12, 1.26]
Mount Alexander	53	29.62	105.60	0.50	0.53 [0.41, 0.67]

Moyne	41	13.08	390.05	0.11	0.13	[0.10, 0.17]
Murrindindi	71	52.89	74.88	0.95	0.95	[0.75, 1.17]
Nillumbik	207	32.99	275.98	0.75	0.76	[0.66, 0.86]
Nth Grampians	45	37.67	69.42	0.65	0.65	[0.49, 0.84]
Port Phillip	453	45.12	449.72	1.01	1.01	[0.92, 1.10]
Pyrenees	29	42.99	40.87	0.71	0.70	[0.49, 0.95]
Queenscliffe	19	61.59	21.97	0.86	0.85	[0.54, 1.25]
Sth Gippsland	88	31.70	156.15	0.56	0.58	[0.47, 0.70]
Sth Grampians	78	47.64	92.10	0.85	0.82	[0.65, 1.00]
Stonnington	399	39.43	495.16	0.81	0.81	[0.73, 0.89]
Strathbogie	54	55.87	61.76	0.87	0.88	[0.67, 1.11]
Surf Coast	77	28.03	134.56	0.57	0.58	[0.46, 0.71]
Swan Hill	132	63.01	106.88	1.24	1.20	[1.01, 1.42]
Towong	15	25.38	35.12	0.43	0.50	[0.32, 0.72]
Wangaratta	99	36.37	150.32	0.66	0.67	[0.55, 0.80]
Warrnambool	156	47.27	166.09	0.94	0.92	[0.78, 1.07]
Wellington	151	35.70	222.74	0.68	0.68	[0.58, 0.79]
West Wimmera	2	4.77	25.32	0.08	0.30	[0.16, 0.48]
Whitehorse	825	51.79	828.36	1.00	1.00	[0.93, 1.06]
Whittlesea	717	42.19	727.36	0.99	0.99	[0.92, 1.06]
Wodonga	141	38.55	164.70	0.86	0.85	[0.71, 0.99]
Wyndham	771	43.11	669.24	1.15	1.15	[1.07, 1.23]
Yarra	522	64.45	354.04	1.47	1.47	[1.34, 1.60]
Yarra Ranges	715	47.91	698.29	1.02	1.02	[0.95, 1.10]
Yarriambiack	22	30.98	45.54	0.48	0.52	[0.35, 0.72]

Table 5: Hyperglycaemia: Observed case rate, standardised case rate and standardised incidence ratio

LGA	*Observed caseload*	*Observed case rate (per 10,000)*	*Expected caseload*	*SIR (crude)*	*SIR [CrI] (smoothed)*
Alpine	23	18.99	30.67	0.75	0.77 [0.52 ,1.07]
Ararat	40	35.29	27.83	1.44	1.31 [0.94 ,1.75]
Ballarat	164	16.92	204.93	0.80	0.80 [0.68 ,0.93]
Banyule	233	18.85	269.62	0.86	0.87 [0.75 ,0.99]
Bass Coast	63	20.58	78.21	0.81	0.81 [0.63 ,1.02]
Baw Baw	50	11.29	97.45	0.51	0.55 [0.41 ,0.70]
Bayside	142	14.64	226.65	0.63	0.64 [0.53 ,0.75]
Benalla	34	24.76	35.24	0.96	0.95 [0.68 ,1.28]
Boroondara	182	10.80	365.61	0.50	0.51 [0.43 ,0.59]
Brimbank	483	24.98	368.60	1.31	1.31 [1.18 ,1.45]
Buloke	19	29.90	17.39	1.09	1.02 [0.65 ,1.48]
Campaspe	79	21.37	87.54	0.90	0.90 [0.71 ,1.11]
Cardinia	131	16.34	144.77	0.90	0.91 [0.75 ,1.07]
Casey	698	26.08	466.24	1.50	1.49 [1.36 ,1.64]
Central Goldfields	36	28.61	33.80	1.07	1.02 [0.72 ,1.37]
Colac-Otway	31	14.93	49.17	0.63	0.64 [0.45 ,0.87]
Corangamite	37	22.58	38.83	0.95	0.91 [0.65 ,1.22]
Darebin	431	29.80	305.47	1.41	1.41 [1.26 ,1.56]
East Gippsland	141	32.67	110.88	1.27	1.25 [1.05 ,1.48]
Frankston	464	35.22	268.25	1.73	1.72 [1.54 ,1.91]
Gannawarra	12	11.57	27.90	0.43	0.53 [0.32 ,0.79]
Glen Eira	219	15.73	302.72	0.72	0.73 [0.63 ,0.83]
Glenelg	53	26.82	45.67	1.16	1.10 [0.82 ,1.42]
Golden Plains	15	7.70	36.21	0.41	0.49 [0.31 ,0.71]
Gr. Bendigo	240	23.16	224.14	1.07	1.07 [0.93 ,1.22]
Gr Dandenong	459	31.81	295.40	1.55	1.55 [1.39 ,1.72]
Gr. Geelong	373	17.10	489.08	0.76	0.76 [0.68 ,0.85]
Gr. Shepparton	125	20.04	131.25	0.95	0.95 [0.79 ,1.13]
Hepburn	15	10.18	35.22	0.43	0.51 [0.32 ,0.74]
Hindmarsh	11	19.03	15.95	0.69	0.72 [0.42 ,1.11]
Hobsons Bay	189	21.41	182.54	1.04	1.04 [0.89 ,1.20]
Horsham	38	19.36	45.63	0.83	0.82 [0.59 ,1.09]
Hume	467	26.23	308.48	1.51	1.51 [1.35 ,1.67]
Indigo	13	8.46	34.92	0.37	0.47 [0.29 ,0.70]
Kingston	290	19.33	330.72	0.88	0.88 [0.77 ,0.99]
Knox	343	22.19	313.01	1.10	1.09 [0.97 ,1.23]
Latrobe	284	38.38	159.45	1.78	1.76 [1.54 ,1.99]
Loddon	20	26.65	20.17	0.99	0.95 [0.62 ,1.36]
Macedon Ranges	44	10.11	88.22	0.50	0.53 [0.40 ,0.69]
Manningham	172	14.70	276.67	0.62	0.63 [0.54 ,0.73]
Mansfield	24	29.81	18.77	1.28	1.17 [0.78 ,1.66]
Maribyrnong	180	23.42	142.58	1.26	1.26 [1.07 ,1.46]
Maroondah	266	24.59	228.62	1.16	1.16 [1.01 ,1.32]
Melbourne	260	24.66	176.14	1.48	1.46 [1.28 ,1.66]
Melton	294	24.94	185.37	1.59	1.57 [1.38 ,1.78]
Mildura	137	26.25	114.85	1.19	1.17 [0.98 ,1.39]
Mitchell	80	22.13	69.31	1.15	1.13 [0.90 ,1.39]
Moira	80	27.93	71.89	1.11	1.09 [0.86 ,1.35]
Monash	294	16.34	405.37	0.73	0.73 [0.64 ,0.82]
Moonee Valley	243	21.41	247.14	0.98	0.99 [0.86 ,1.12]
Moorabool	57	19.42	58.13	0.98	0.96 [0.73 ,1.22]
Moreland	482	30.77	333.46	1.45	1.44 [1.30 ,1.60]
Mornington	382	25.33	374.66	1.02	1.02 [0.91 ,1.14]
Mount Alexander	14	7.82	45.10	0.31	0.41 [0.26 ,0.59]

Moyne	22	7.02	197.68	0.11	0.16	[0.11 ,0.22]
Murrindindi	18	13.41	31.34	0.57	0.64	[0.42 ,0.92]
Nillumbik	64	10.20	115.60	0.55	0.58	[0.45 ,0.72]
Nth Grampians	31	25.95	29.93	1.04	0.99	[0.69 ,1.35]
Port Phillip	175	17.43	187.87	0.93	0.93	[0.79 ,1.08]
Pyrenees	12	17.79	17.26	0.70	0.72	[0.44 ,1.10]
Queenscliffe	6	19.45	9.77	0.61	0.68	[0.33 ,1.17]
Sth Gippsland	57	20.54	66.95	0.85	0.86	[0.65 ,1.09]
Sth Grampians	22	13.44	40.23	0.55	0.58	[0.39 ,0.82]
Stonnington	136	13.44	214.64	0.63	0.64	[0.54 ,0.76]
Strathbogie	29	30.00	26.53	1.09	1.05	[0.73 ,1.44]
Surf Coast	21	7.64	56.74	0.37	0.42	[0.28 ,0.59]
Swan Hill	42	20.05	46.09	0.91	0.90	[0.66 ,1.18]
Towong	13	22.00	14.98	0.87	0.86	[0.52 ,1.31]
Wangaratta	38	13.96	65.16	0.58	0.61	[0.45 ,0.81]
Warrnambool	68	20.60	72.60	0.94	0.92	[0.71 ,1.15]
Wellington	49	11.59	95.23	0.51	0.55	[0.41 ,0.70]
West Wimmera	2	4.77	10.85	0.18	0.47	[0.23 ,0.81]
Whitehorse	243	15.25	363.04	0.67	0.67	[0.59 ,0.77]
Whittlesea	344	20.24	308.26	1.12	1.11	[0.99 ,1.25]
Wodonga	62	16.95	70.87	0.87	0.87	[0.67 ,1.10]
Wyndham	303	16.94	281.63	1.08	1.07	[0.95 ,1.21]
Yarra	167	20.62	150.08	1.11	1.11	[0.94 ,1.29]
Yarra Ranges	257	17.22	295.86	0.87	0.87	[0.76 ,0.99]
Yarriambiack	12	16.90	19.92	0.60	0.66	[0.40 ,1.01]

Table 6: Factors associated with risk of prehospital EMS attendance for hypoglycaemia: Unadjusted models

Area-level factor	RR	95% CrI	DIC
**Ethnicity (percentage of oversea-born residents)**			712.67
1 (low proportion overseas-born residents)	Reference		
2	1.35	[1.00, 1.81]	
3	1.68	[1.23, 2.30]*	
4	1.92	[1.35, 2.71]*	
5 (high proportion overseas-born residents)	2.17	[1.51, 3.06]*	
**Access to motor vehicle**			713.79
1 (most access to motor vehicle)	Reference		
2	1.07	[0.81, 1.41]	
3	1.12	[0.85, 1.48]	
4	1.54	[1.16, 2.04]*	
5 (least access to motor vehicle)	1.54	[1.15, 2.08]*	
**IEO (education and occupation)**			712.82
1 (least education)	Reference		
2	0.85	[0.64, 1.13]	
3	0.83	[0.63, 1.10]	
4	0.66	[0.49, 0.88]*	
5 (most education)	0.67	[0.47, 0.96]*	
**IRSD (socioeconomic disadvantage)**			714.17
1 (most disadvantage)	Reference		
2	0.78	[0.58, 1.03]	
3	0.81	[0.61, 1.09]	
4	0.77	[0.56, 1.06]	
5 (least disadvantage)	0.68	[0.47, 0.97]*	
**IRSAD (socioeconomic advantage & disadvantage)**			713.67
1 (most disadvantage)	Reference		
2	0.78	[0.58, 1.05]	
3	0.79	[0.59, 1.06]	
4	0.66	[0.48, 0.91]*	
5 (most advantage)	0.66	[0.45, 0.96]*	
**IER (economic resource)**			714.83
1 (least wealth)	Reference		
2	0.76	[0.57, 1.01]	
3	0.72	[0.55, 0.95]*	
4	0.73	[0.55, 0.97]*	
5 (most wealth)	0.58	[0.43, 0.78]*	
**ARIA (remoteness)**			714.92
1 (major city)	Reference		
2	0.89	[0.66, 1.20]	
3	0.70	[0.50, 0.98]*	
4	0.60	[0.43, 0.86]*	
5	0.80	[0.51, 1.32]	
6 (remote)	0.64	[0.38, 1.13]	
**Population density (residents per km^2)**			714.44
1 (low density)	Reference		
2	0.89	[0.66, 1.21]	
3	1.12	[0.80, 1.54]	
4	1.47	[0.99, 2.11]	
5 (high density)	1.45	[0.95, 2.14]	

**Prevalence of diabetes (percentage residents with diabetes)**			714.55
Q1 (low prevalence)	Reference		
Q2	0.79	[0.56, 1.10]	
Q3	0.92	[0.68, 1.24]	
Q4	1.01	[0.73, 1.40]	
5 (high prevalence)	0.97	[0.70, 1.36]	

* Indicates statistical significance (i.e. 95%CrI does not cross 0)

Table 7: Factors associated with risk of prehospital EMS attendance for hypoglycaemia: Multivariable model

Area-level factor	RR	95% CrI	DIC
			712.03
**Ethnicity (percentage of oversea-born residents)**			
1 (low proportion overseas-born residents)	Reference		
2	1.35	[1.02, 1.79]*	
3	1.74	[1.28, 2.35]*	
4	1.83	[1.28, 2.56]*	
5 (high proportion overseas-born residents)	2.04	[1.39, 2.91]*	
**Access to motor vehicle**			
1 (most access to motor vehicle)	Reference		
2	1.10	[0.84, 1.43]	
3	1.18	[0.91, 1.53]	
4	1.56	[1.20, 2.03]*	
5 (least access to motor vehicle)	1.38	[1.04, 1.83]*	

* Indicates statistical significance (i.e. 95%CrI does not cross 0)

Table 8: Factors associated with risk of prehospital EMS attendance for hyperglycaemia: Unadjusted models

Area-level factor	SIR	95% CrI	DIC
**Ethnicity (percentage of oversea-born residents)**			642.07
1 (low proportion overseas-born residents)	Reference		
2	1.25	[0.89, 1.74]	
3	1.20	[0.84, 1.71]	
4	1.51	[1.02, 2.34]*	
5 (high proportion overseas-born residents)	1.81	[1.22, 2.80]*	
**Access to motor vehicle**			641.04
1 (most access to motor vehicle)	Reference		
2	1.30	[0.95, 1.77]	
3	1.17	[0.86, 1.59]	
4	1.53	[1.12, 2.10]*	
5 (least access to motor vehicle)	1.55	[1.13, 2.14]*	
**IEO (education and occupation)**			641.60
1 (least education)	Reference		
2	0.86	[0.65, 1.14]	
3	0.79	[0.60, 1.04]	
4	0.56	[0.42, 0.75]*	
5 (most education)	0.48	[0.33, 0.68]*	
**IRSD (socioeconomic disadvantage)**			642.96
1 (most disadvantage)	Reference		
2	0.71	[0.54, 0.95]	
3	0.75	[0.56, 1.00]	
4	0.65	[0.48, 0.89]*	
5 (least disadvantage)	0.47	[0.33, 0.66]*	
**IRSAD (socioeconomic advantage & disadvantage)**			642.58
1 (most disadvantage)	Reference		
2	0.82	[0.61, 1.10]	
3	0.70	[0.52, 0.94]*	
4	0.64	[0.46, 0.88]*	
5 (most advantage)	0.47	[0.32, 0.68]*	
**IER (economic resource)**			642.17
1 (least wealth)	Reference		
2	0.83	[0.61, 1.13]	
3	0.69	[0.52, 0.92]*	
4	0.65	[0.48, 0.87]*	
5 (most wealth)	0.55	[0.40, 0.75]*	
**ARIA (remoteness)**			642.60
1 (major city)	Reference		
2	0.96	[0.68, 1.34]	
3	0.67	[0.43, 0.97]*	
4	0.67	[0.43, 0.99]*	
5	0.91	[0.53, 1.55]	
6 (remote)	0.78	[0.42, 1.40]	
**Population density (residents per km^2)**	Reference		642.80
1 (low density)	0.80	[0.58, 1.12]	
2	0.88	[0.62, 1.26]	
3	1.27	[0.85, 1.90]	
4	1.08	[0.70, 1.69]	
5 (high density)			

**Prevalence of diabetes (percentage residents with**	Reference		642.71
**diabetes)**	1.03	[0.73, 1.43]	
Q1 (low prevalence)	1.22	[0.89, 1.66]	
Q2	1.31	[0.94, 1.83]	
Q3	1.59	[1.13, 2.25]*	
Q4			
5 (high prevalence)			

* Indicates statistical significance (i.e. 95%CrI does not cross 0)

Table 9: Factors associated with risk of prehospital EMS attendance for hyperglycaemia: Multivariable model

Area-level factor	SIR	95% CrI	DIC
			641.36
**Ethnicity (percentage of oversea-born residents)**			
1 (low proportion overseas-born residents)	Reference		
2	1.15	[0.84, 1.58]	
3	1.26	[0.89, 1.80]	
4	1.82	[1.24, 2.65]*	
5 (high proportion overseas-born residents)	1.90	[1.27, 2.80]*	
**IRSD (socioeconomic disadvantage)**			
1 (most disadvantage)	Reference		
2	0.88	[0.65, 1.22]	
3	0.85	[0.59, 1.25]	
4	0.78	[0.49, 1.26]	
5 (least disadvantage)	0.55	[0.34, 0.91]*	

* Indicates statistical significance (i.e. 95%CrI does not cross 0)
